# Mutational analysis of hemoglobin genes and functional characterization of detected variants, through in-silico analysis, in Pakistani beta-thalassemia major patients

**DOI:** 10.1038/s41598-023-35481-1

**Published:** 2023-08-14

**Authors:** Samina Ejaz, Iqra Abdullah, Muhammad Usman, Muhammad Arslan Iqbal, Sidra Munawar, Muhammad Irfan Khan, Nagina Imtiaz, Hanniah Tahir, Muhammad Ihsan Bari, Tayyaba Rasool, Aneeza Fatima, Ramsha Anwar, Ayman Durrani, Yasir Hameed

**Affiliations:** 1 Department of Biochemistry, Institute of Biochemistry, Biotechnology and Bioinformatics (IBBB), The Islamia University of Bahawalpur, Bahawalpur, 63100 Pakistan; 2 Department of Biotechnology, Institute of Biochemistry, Biotechnology and Bioinformatics (IBBB), The Islamia University of Bahawalpur, Bahawalpur, 63100 Pakistan; 3 Punjab Institute of Neurosciences, Lahore, Pakistan; 4 Department of Hematological Diseases, Thalassemia and Bone Marrow Transplantation, Bahawal Victoria Hospital, Bahawalpur, 63100 Pakistan

**Keywords:** Biochemistry, Biotechnology, Computational biology and bioinformatics, Diseases, Health care

## Abstract

Thalassemia is one of the most prevalent genetic disorders worldwide. The present study aimed to explore the mutational spectrum of all hemoglobin (HB) encoding genes and to identify the potentially damaging and pathogenic variants in the beta (β)-thalassemia major patients and thalassemia minor carriers of Southern Punjab, Pakistan. A total of 49 β-thalassemia major patients and 49 carrier samples were screened for the identification of HBA1, HBA2, HBB, HBD, HBE1, HBG1 and HBG2 variants by NGS. PCR was performed for the amplification of HB encoding genes and the amplified product of 13 patients and 7 carrier samples were processed for the Sanger sequencing. Various bioinformatics tools and databases were employed to reveal the functional impact and pathogenicity potential of the observed variants. Results depicted a total of 20 variants of HB-related genes by NGS and 5 by Sanger sequencing in thalassemia patients. While 20 variants by NGS and 3 by Sanger were detected in carriers. Few known genetic variants of HB-encoding genes are being reported for the first time in Pakistani thalassemia patients and carriers. However, two novel HBB variants c.375A>C (p.P125P) and c.*61T>G and a novel variant of HBE1 (c.37A>T (p.T13S)) were also documented. Pathogenicity analysis predicted the pathogenic potential of HBB variants (c.47G>A (p.W16*), c.27-28insG (p. S10fs), and c.92+5G>C) for β thalassemia. The study of functional impact indicated that these HBB variants result in the premature termination of translation leading to the loss of functional β-globin protein. It is therefore suggested that the pathogenic HBB variants, identified during present study, can be employed for the diagnosis, carrier screening, and planning therapy of thalassemia.

## Introduction

Thalassemia is an inherited hematological disorder of hemoglobin synthesis resulting from an aberration in the hemoglobin (Hb) encoding genes^1^. The Hb is a tetrameric molecule present in the erythrocytes which is composed of four polypeptide chains of globin and transports oxygen in the body. Each globin chain is encoded by a specific globin gene and types of globin chains determine the type of HB. Adult Hb (HbA) is the most common Hb comprising two β-chains and two α-chains^2^. In thalassemia reduced synthesis or complete absence of globin chains is observed. Depending on the defective globin chain thalassemia is classified into two main types, α-thalassemia and β-thalassemia^3^. Prevalence of thalassemia is reported to be highest in the Middle East, Mediterranean region, and Southeast Asia populations^4^. In Pakistan thalassemia is prevalent in the areas of Sindh and Balochistan provinces where intermarriages are more common^5^. Thalassemia has become a major health issue due to high carrier rates. Worldwide in more than 60 countries, the carrier rate of β-thalassemia has been estimated to be 150 million while in Pakistan, the β-thalassemia carrier rate has reached up to 7%, and annually more than 9000 children are born with homozygous β-thalassemia^6^. At present 1407 variants of Hb encoding genes (HBA, HBB, HBD, and HBG) have been reported in the Hbvar database (https://globin.bx.psu.edu/hbvar/menu.html). All these HB variants are responsible for the production of defective globin. In Pakistan, 90% of thalassemia genotypes are associated with the six most commonly occurring variants of the β-globin gene including HBB:c.27_28insG(p.Ser10Valfs*14), HBB:c.92+5G>C, HBB:c.47G>A(p.Trp16*), HBB:c.9+1G>T, HBB:c.126_129delCTTT(p.Phe42Leufs*19) and NG_000007.3:g.71609_72227del619^7^. Hence, the majority of researchers have focused to screen Pakistani thalassemia patients for these HBB variants and 3 deletional variants of the HBA gene including − α 3.7 (a common deletion variant related to alpha thalassemia), − α 4.2 and ααα anti-3.7 i.e. a rare variants^8^. Although in Pakistani thalassemia patients the variant profiling has been limited to the HBB gene^9^, globally variants in other globin genes have also been examined and found to be involved in the development of thalassemia^10^. Similarly none of the studies conducted in Pakistan has reported the functional effect and the pathogenicity potential of the Hb gene variants. To fill in the existing gap this study was planned to screen the whole genome of thalassemia patients and carriers of Southern Punjab, Pakistan by NGS analysis for the detection of variants, if any, in all the Hb-related genes. The NGS data was further subjected to bioinformatics analysis to identify the pathogenic and disease-causing variants among the detected variants. The information thus generated would pave the way for better thalassemia diagnosis, screening, genetic counseling, and control.

Human Hb is mainly comprised of adult HbA (95–98%) and a small amount of HbA2 (2–3%) and HbF (0.8 to 2%). HbA consists of 2 alpha and 2 β chains and is referred to as α2β2 tetramer. While HbA2 is composed of two α chains and two δ chains and HbF is an α2γ2 tetramer. The genetic alteration in any of the globin genes leads to the reduction in the quantity of one globin chain and a comparative increment in other chains. This is a situation that prevails in a disorder like thalassemia. Hemoglobin A2 is a useful indicator for screening of thalassemia carriers. The elevated level of hemoglobin A2 (HbA2) is proven as the most significant feature of β-thalassemia carriers. Hence, the HbA2 level is proposed to be a good biomarker that can be used for screening β-thalassemia trait in pregnant women in the South China population^11^. Hemoglobin A2 (HbA2) levels>3.5% are associated with β-thalassemia and < 2.5% is the feature of α-thalassemia^12,13^. A recent study identified HbA2 an efficient indicator for detecting intermediate types of thalassemia including α-, β-, and αβ compound thalassemia. Moreover, it was concluded that combined measurement of HbA2 and HbF is the more effective strategy for detecting carriers for β-thalassemia variants^14^.

Knowing the importance of globin genes other than the one encoding β chain of Hb (i.e., α, γ, δ encoding genes) this research project was planned to investigate the mutational spectrum of all Hb-related genes in Pakistani β-Thalassemia major patients and the suspected carriers. The presence of other globin genes’ variants and their dysregulated expression has been linked with the severity of disease in patients and carrier status confirmation in a population. It was therefore speculated that the mutational profile of all the Hb- related genes will lead to improving the clinical conditions and better management of disease in patients. The information thus generated can also help to lay down the basis of genetic counseling for couples at risk for thalassemia major.

## Methodology

### Ethical committee approval

All the experiments and protocols were carried out in accordance with relevant guidelines and regulations approved by Helsinki convention 1992. This study was approved by the 10th Meeting of the Board of Studies (an IRB) of the Department of Biochemistry and Biotechnology held on 4th February 2020 (Approval granted via letter No. 1064/ BBT). Informed consent was obtained from all subjects and their legal guardian(s).

### Patients’ enrollment in the study

A total of 49 clinically diagnosed blood transfusion-dependent β-thalassemia major patients including male (n = 28) and female (n = 21), and 49 thalassemia minor trait carriers (males: n = 31 and females: n = 18) referred from the Thalassemia center of Bahawalpur Victoria Hospital, Bahawalpur took part in the present study. Blood transfusion-dependent thalassemia major patients of age ranging from 6 months to 15 years and having blood transfusion interval of only 15–30 days were enrolled in this study followed by the consent of parents or guardians. The parents signed the consent letter and agreed to donate blood samples. While non-blood transfusion dependent thalassemia patients, patients suffering from hepatitis and whose parents refused to participate were excluded. The carriers were identified using the cut-off value MCV (fL) < 80 and MCH (pg) < 27 as referred by previous studies^15,16^. The detailed demographic and clinical report of patients has been provided in Supplementary Table S1 and carriers demography and detailed information of patients and has been given in Supplementary Tables S2 and S3, respectively.

### Genomic DNA extraction

Genomic DNA was isolated from whole blood using an already reported protocol (Sambrook and Russell, 2006). Extracted DNA was quantified by the Nano-drop method (Biotek/Take3) and the quality of extracted DNA was visualized on 1% agarose gel via electrophoresis. The extracted DNA was stored at -80˚C till further analysis.

### Next generation sequencing (NGS)

The two consortiums of diseased samples (n = 49) and carrier samples (n = 49) were prepared separately by adding DNA volume having equimolar concentration (ng/µl) i.e. 150 of each DNA sample. The samples were sent to Macrogen, South Korea for whole genome sequencing by NGS analysis. The total size (bp) of the targeted sequences was 24,736,809 for the carrier samples’ consortium and 23,980,170 for the diseased consortium. Analysis of NGS results, obtained in FASTQ files, was performed using different bioinformatics softwares. The FASTQC software was used for the assessment of sequence read quality and low-quality reads were removed using Trimmomatic (version 0.32). Reads were then mapped to the human reference genome GRCh38 using Burrows-Wheeler Aligner (BWA) version 0.6.2 software. The sorting and indexing of BAMs files were done using SAMtools. The duplications of reads were removed by Picard (http://picard.sourceforge.net). Variant calling was done with GATK Unified Genotyper (https://gatk.broadinstitute.org) which employs a variant filtration program to filter data for allele balance (AB>0.75), quality score (QUAL10) and mapping quality zero reads (MQ0 ≥ 4). The BCF tools were also used for SNP and Indel calling and in initiating VCFs file that was merged using VCF tools and indexed using Tabix. Variants were annotated by SnpEff (version 4.3) tool.

### Validation by Sanger sequencing

For the validation of NGS results out of 98 samples (49 thalassemia samples and 49 carrier samples) a total of 13 thalassemia patients’ samples and only 7 carrier samples (due to funding limitations) were subjected to PCR amplification of all Hb-related genes and Sanger sequencing of the amplified products. The primers for all the Hb encoding genes were designed using Primer-3 (version 0.4.0) an online tool available at: https://bioinfo.ut.ee/primer3-0.4.0/ (Supplementary Table S4). A total of 50 µl volume of PCR reaction mixture, containing 50 ng of template DNA, 20 pmol of forward and reverse primers, 1.25 U of Taq DNA polymerase (ThermoFisher Scientific, Boston, MA, USA), 200 µM dNTPs, 2 mM of MgSO_4_ and 1X PCR buffer, was used. PCR amplification was performed by the Mygene TmL series Peltier thermal cycler (UNIEQUIP). For amplification initial denaturation was carried out at 95 °C for 5 min followed by 40 cycles of denaturation at 94 °C for 30 s, annealing at a specific temperature for each gene for 30s and extension at 72 °C for 1 min. The final extension was performed for 10 min at 72 °C. The quality of PCR products was confirmed by 2% agarose gel electrophoresis. The amplified product was purified using Thermofisher purification kit (Cat#T1030S) and the PCR amplicons were sent to Lab Genetix, Pakistan for Sanger sequence analysis.

### Sanger sequencing results analysis

Sanger sequencing results were evaluated by comparative and variant analysis. In a comparative analysis, gene sequences (wild type) were retrieved from NCBI, National Center of Biotechnology (https://www.ncbi.nlm.nih.gov/gene/3043). To determine the sequence similarity across all genomes BLAST tool (https://blast.ncbi.nlm.nih.gov/Blast.cgi) was used. Next, variants were identified using the Pairwise sequence alignment tool (https://www.ebi.ac.uk/Tools/psa/emboss_needle/) by comparing the retrieved sequences and the wild-type gene sequences. The EXPASY translator tool (https://web.expasy.org/translate/) was employed for the translational of the retrieved exonic sequences into protein sequences to determine any change in the protein sequence due to variants.

### In-silico analysis

The functional characterization and pathogenicity of detected variants were explored using the various bioinformatics tools. Functional annotation of all the detected synonymous and non-synonymous variants was performed using the Mutation Taster tool which helped to study the effect of observed variants on the splice site, on the protein function and to predict their role in disease. The impact of missense and silent variants was further confirmed using various freely available tools including PROVEAN (http://provean.jcvi.org/index.php), SNAP2 (https://rostlab.org/services/snap2web/), Polyphen2 (http://genetics.bwh.harvard.edu/pph2/), SIFT (https://sift.bii.a-star.edu.sg/) and PANTHER (http://www.pantherdb.org/tools/). Human splicing Finder (HSF) version 3.1 (http://umd.be/Redirect.html) was used to deduce the functional impact of exonic and intronic variants on the splicing pattern and splice sites i.e. splice donor site, branch point, splice acceptor site, splicing enhancer and splicing silencer motifs. The score 65 was considered as a threshold value for HSF. While the effect of 3′ UTR variants on the miRNA binding sites was explored by PolymiRT (https://compbio.uthsc.edu/miRSNP/) database. Protein stability of missense variants was instigated by the online tool I-mutant (http://gpcr2.biocomp.unibo.it/cgi/predictors/I-Mutant3.0/I-Mutant3.0.cgi) and Mupro (http://mupro.proteomics.ics.uci.edu/). Varsome (https://varsome.com/) was used as a major tool for predicting pathogenicity. It uses the ACMG-AMP recommendation and standards to classify the variants to be benign or pathogenic. The Phyre 2 (http://www.sbg.bio.ic.ac.uk/phyre2/html/page.cgi?id=index) web server predicted the missense variant effect on the secondary and 3-Dimensional structure of the mutant protein.

## Results

### NGS analysis results

In our study, by NGS analysis 20 variants in thalassemia patients and 20 variants in carriers were detected collectively for all the Hb-encoding genes (Table 1).

**Table 1:** Variants identified by NGS analysis and Sanger sequencing in thalassemia patients and carriers.

Sr. no	Genes	Detected by NGS analysis		Detection by Sanger sequencing	β Thalassemia phenotype			Frequencies (%) detected by NGS		Validation by database	
		Location	Variants		In patients	In carriers	Variant phenotype	In patients	In carriers	Hbvar	dbSNP
1	HBA1	176,693	**c.-24C>G**	–	Major	Minor		50	50	Nil	rs374054030
2	HBA2	173,707	c.*107A>G	–	Major	Minor		100	100	1177	rs2541640
		173,736	c.*136A>G^Ϯ^	–	Major	–		100	–	Nil	rs2685121
		172,889	c.-24C>G^#^	–	–	Minor		–	100	2927	rs772829778
3	HBB	5,226,561	**c.315**+**16G>C**	+ (in patient)	Major	Minor	(β^+^) SNP marker	50	50	3045	rs10768683
		5,227,013	**c.9T>C(p.H3H)**	–	Major	Minor	β	50	50	3042	rs713040
		5,225,911	**c.316-185C>T**	+ (in patient)	Major	Minor	(β^+^) SNP marker	50	50	3050	rs1609812
		5,226,503	**c.315**+**74T>G**	+ (in patient)	Major	Minor	(β^+^) SNP marker	50	50	2930	rs7480526
		5,226,975	c.47G>A(p.W16*)^Ϯ^	–	Major	–	β^0^	50	–	791	rs63750783
		5,226,994	c.27-28insG(p.S10fs) ^#^	–	–	Minor	β^0^	–	50	786	rs35699606
		5,226,925	c.92+5G>C ^Ϯ^	–	Major	–	β^+^	50	–	824	rs33915217
4	HBE1	5,268,666	**c.316-85insGTTT**	–	Major	Minor		50	50	Nil	rs3061750
5	HBG1	5,248,576	**c.316-89G>T**	–	Major	Minor		100	50	Nil	rs28379094
		5,248,304	c.*55delA	–	Major	Minor		50	50	Nil	rs3841756
		5,248,353	c.*6delC	–	Major	Minor		50	50	Nil	rs757054403
		5,248,354	c.*5A>T	–	Major	Minor		50	50	Nil	rs1065686
		5,248,356	c.*2dupC	–	Major	Minor		50	50	Nil	rs371890964
		5,248,569	**c.316**–**82T>G**	–	Major	Minor		100	50	Nil	rs28440105
		5,249,309	**c.315**+**59G>T**	–	Major	Minor		50	50	Nil	rs33988501
6	HBG2	5,253,222	c.*54_*55insA^#^	–	–	Minor		–	50	Nil	rs34879481
		5,254,177	**c.315**+**115A>T**	–	Major	Minor		50	50	Nil	rs2070973
		5,254,268	**c.315**+**24A>C**	–	Major	Minor		50	50	Nil	rs33993529
		5,254,572	**c.93-58C>T**	–	Major	Minor		50	50	Nil	rs1894398

Variants highlighted in bold were the common variants detected in both thalassemia patients and carriers.

^#^Represents variants found in carrier samples. ^Ϯ^represents variants detected only in thalassemia patients’ samples. − represents variants not detected. *Nil* not reported by HbVar.

### HBA1 and HBA2 variants

In HBA1 a 5′ untranslated region (UTR) variant HBA1: c.-24C>G was found to be present in both the patients and carrier samples consortium. For HBA2, 2 non-deletion variants c.*107A>G and c.*136A>G in the 3′ UTR were identified in the patients. While a 3′ UTR variant c.*107A>G and a 5′ UTR c.-24C>G variant were noticed in the carriers. Previously, these variants were reported neither in Pakistani thalassemia patients nor in the carriers.

### HBB variants

Variant analysis of HBB by NGS revealed 6 variants comprising two exonic variants i.e., a silent variant c.9T>C (p.H3H) observed at CD2 and a non-sense variant c.47G>A (p.W16*) at CD15 which results in the formation of a truncated protein, one 5′ splice site variant c.92+5G>C and three intronic variants c.316-185C>T, c.315+74T>G, c.315+16G>C were detected in thalassemia patients. While in carriers, 5 variants including two exonic variants namely frameshift variants c.27-28insG (p.S10fs) at CD 8/9, a silent variant c.9T>C (p.H3H) at CD2, and three intronic variants including c.316-185C>T, c.315+74T>G and c.315+16G>C were identified. The frameshift variant c.27-28insG resulted in the change in the amino acid sequence in the downstream ORF. Of these variants, 4 were found in both thalassemia patients and carriers.

### HBD and HBE1 variants

In the present study, no HBD variant was detected either in thalassemia patients or carriers by NGS analysis. Only one intronic HBE1 variant c.316-85insGTTT was noted for the first time in both the Pakistani patients and carriers’ samples.

### HBG1 and HBG2 variants

Results exhibited that HBG1 gene harbors 7 similar variants in both the thalassemia patients and the carriers including 3 intronic variants (c.316-82T>G, c.315+59G>T and c.316-89G>T) and 4, 3′ UTR variants (c.*55delA, c.*6delC, c.*5A>T and c.*2dupC). For HBG2, overall, 4 variants including 3 intronic variants, c.315+24A>C, c.315+115A>T and c.93-58C>T, were detected in both the patients and carriers. While a 3' UTR c.*54_*55insA variant was noticed only in the carrier samples. All these HBG1 and HBG2 variants have not been reported earlier in the Pakistani population.

### Variants validation by Sanger sequencing

Sanger sequencing ascertained variants only in HBB and HBE1 genes. The three HBB variants (c.315+74T>G, c.316-185C>T, and c.315+16G>C) detected by NGS were confirmed by Sanger sequencing in two thalassemia patients (Table 1). Apart from NGS validation, Sanger sequencing also manifested two novel variants (reported for the first time worldwide) in the HBB gene i.e., a silent variant c.375A>C (p.P125P) at CD 124 and c.*61T>G variants. While a unique (reported for the first time in Pakistan) missense variant c.386C>A (p.A129D) at CD 128 in one carrier sample (sample ID: N15). Besides these, in HBE1 one novel missense variant at codon 12 (c.37A>T) which results in the alteration of amino sequence T13S and a unique splice site variant c.92+5(G>A) was discovered in only one patient sample (Table 2).

**Table 2:** Novel and unique variants detected by only Sanger sequencing.

Sr. no	Gene	Location	Variants	β Thalassemia Phenotype			HbVar ID
				In patients	In Carriers	Variant phenotype	
1	HBB	5,225,656	c.386C>A(p.A129D)	–	Minor	Mild β^+^	525
		5,225,667	c.375A>C(p.P125P)^ϕ^	–	Minor	Mild β^+^	NIL
		5,225,537	c.*61T>G^ϕ^	–	Minor		NIL
2	HBE1	5,269,854	c.37A>T(p.T13S)^ϕ^	Major	–		NIL
		5,269,795	c.92+5G>A	Major	–		NIL

Φ = novel variants, – represents variants not detected, *Nil* not reported by HbVar.

### Functional impact of exonic variants

In silico analysis was performed to determine the impact of the amino acid changes on the protein function and stability. The impact of HBB missense variant c.386C>A(p.A129D), anticipated through MutationTaster and PredictSNP, revealed this variant to be disease-causing resulting in the loss of helix (125–143 amino acid position) and deleterious with 76% confidence. The high functional impact (FI score = 4.305) of this variant was observed by the Mutation assessor. Moreover, it also seems to decrease the stability of globin protein (DDG value = − 0.54) when tested with I-Mutant and to increase the stability (DDG value = 0.05) when checked by Mupro. The HBB nonsense variant c.47G>A (p.W16*) and frameshift variant c.27-28insG (p.S10Vfs*14) were examined to be disease-causing and deleterious (score = -8.1) by MutationTaster and PROVEAN respectively. Results of PANTHER, FATHMM, PROVEAN and SWIFT revealed that HBB silent variants c.9T>C (p.H3H) and c.375A>C(p.P125P) can be characterized as neutral and tolerated. While the MutationTaster exhibited c.375A>C(p.P125P) to be a disease-causing variant based on its altering effect on splicing pattern. The missense variant of HBE1 (T13S) was found to be a benign variant and neutral with 83% prediction accuracy by PredictSNP. While the MutationTaster revealed this variant as a polymorphism which might affect the protein function i-e loss of helix (6–18 position). Stability indexing showed a decrease in protein stability (DDG value = − 0.51 and − 0.04, respectively) as tested by I-Mutant and Mupro, respectively. Further, the impact on the splicing pattern predicted by Human splicing Finder (HSF) proposed an alteration in the splicing pattern by only two exonic variants of HBB namely c.375A>C(p.P125P) and c.47G>A (p.W16*) resulting in the activation of cryptic splice site and alteration of ESE/ESS motif ratio, respectively. Detailed information has been summarized in Table 3.

**Table 3:** Functional impact analysis of Hb-genes exonic variants via bioinformatics tools.

Genes	Variants	Impact of amino acid alteration							Impact on splicing mechanism		Impact on protein stability			
		Mutation Taster	PROVEAN	SNAP2	Polyphen2	SWIFT	PANTHER	PredictSNP	HSF		I-MUTANT		MUPRO	
		Impact	Impact	Impact	Impact	Impact	Impact	Impact	Impact	Variation Score	Solubility	DDG value	Solubility	DDG value
HBB	c.386C>A (p.A129D)	Disease causing	Deleterious − 4.16	Effect − 19	Probably damaging	Deleterious	Probably damaging	Deleterious 76%	Nil		Decrease	− 0.54	Increase	0.05
	c.47G>A (p.W16*)	Disease causing							Significant alteration in the ESE/ESS motif ratio	− 4				
	c.27-28insG (p.S10fs)	Disease causing	Deleterious − 8.1											
	c.9T>C (p.H3H)	Homozygous Polymorphism	Neutral 0	Neutral − 94		Tolerated	Neutral							
	c.375A>C (p.P125P)	Disease causing	Neutral 0	Neutral − 88		Tolerated	Neutral		Activation of a cryptic Acceptor site	2>3.74 = >87%)				
HBE1	c.37A>T (p.T13S)	Polymorphism	Neutral − 1.45	Neutral − 39	Benign	Tolerated	Probably damaging	Neutral 83%			Decrease	− 0.51	Decrease	− 0.04

PROVEAN detects the impact of amino acid substitution on protein function. The threshold value is − 2.5. A variant having a score > − 2.5 is considered “deleterious” and below this value is referred to as “neutral”. For SNAP2 the cut-off value is 1. The variant with a score above 1 should be considered “effect” and below 1 will be “neutral”. Polyphen 2 predicts the variant’s impact on the protein structure and function based on sequence homology. The cut-off value for a variant to be probably damaging is > 0.98 and to be benign variant is 0.446 if the value is < 0.98 and > 0.46 prediction will be possibly damaging. For SIFT, the cut-off value of prediction is 0.5. A substitution with a score > 0.5 will be tolerated and < 0.5 will be deleterious. PANTHER uses the HMM for predictions. The cut-off value for a variant to be damaging is 0.5. The HSF threshold value is set to be 65 while 3 for MaxEnt. For the existence of a possible new splice site the difference in score should be >  + 3. The DDG value < − 0.5 represents that variant greatly destabilizes the protein, DDG value > 0.5 shows variant increases the stability and DDG <  = 0.5 or >  = − 0.5 shows weak effect on protein stability.

### Functional impact of intronic variants

The functional characterization of intronic variants by Human Splicing Finder (HSF) manifested only 2 variants affecting the splicing pattern i.e., HBB: c.92+5G>C variant which causes the wild type (WT) donor site to be broken (variation score = 86.61>76.6 (− 11.56%) resulting in the alteration of splicing and HBG2: c.315+24A>C variant potentially affecting the splicing by activating a new acceptor splice site (variation score = 4.06>5.88 (44.83%) (Table 4). Rest of the intronic variants, HBB (c.315+74T>G, c.315+16G>C, c.316-185C>T), HBG1 (c.316-89G>T, c.315+59G>T, c.316-82T>G), HBG2 (c.93-58C>T, c.315+24A>C, c.315+115A>T) and HBE1 (c.316-85insGTTT, c.92+5G>A),were examined to have no potential impact on the splicing signals (Data not shown in Table 4).

**Table 4:** Functional impact analysis of Hb-genes intronic variants by HSF.

Sr. no	Gene	Variant	Type	Position	Impact	Variation
1	HBB	c.92 + 5G > C	Donor site	5,226,932	Broken WT Donor Site	86.61 > 76.6 (− 11.56%)
2	HBG2	c.315 + 24A > C	MaxEnt Acceptor site	5,254,274	New Acceptor splice site activation	4.06 > 5.88 (44.83%)

The HSF threshold value is set to be 65 while 3 for MaxEnt. For a broken splice site, the score difference is <  + 10% for HSF and the site is said to be above threshold. MaxEnt score >  + 30% represents activation of a new splice site.

### Functional impact of untranslated region (UTR) variant

The functional impact of 3′ UTR variants on the miRNA target site predicted by PolymiRT shows that out of 8 detected 3′ UTR variants only 2 variants namely HBG1: c.*55delA and HBG2: c.*54_*55insA variants were observed to be in the miRNA target sites but no functional impact of these INDEL variants was observed (Table 5).

**Table 5:** Functional impact analysis of 3′ UTR variants by PolymiRT database.

Sr. no	Gene	Variants	Ancestral allele	Allele	miR ID	miRsite	Functional class	Context score
1	HBG1	c.*55delA	–	A	hsa-miR-137	AGCAAT**A**caaata	O	− 0.238
				–	–	–	–	–
2	HBG2	c.*54_*55insA	–	–	has-miR-126- 5p has-miR 4795-3p	aatcaA**A**TAATAa aatcaAATAATAA	O	0.009 0.016
				A	–	–	–	–

The red highlighted nucleotide in the miRsite sequence is the deleted nucleotide while the bases written in the capital letters are complementary to the miRNA seed region. Functional class O represents that the ancestral allele cannot be identified. Context score: an increase in negative score indicates increased functionality. The "*" represents no specific effect of indel variants.

### Pathogenicity prediction of detected HB genes variants

Interpretation of all the exonic, intronic and UTRs variants by Varsome exhibited that only 3 HBB variants including c.47G>A (p.W16*), c.92+5G>C and c.27-28insG (p.S10fs) were predicted to be Pathogenic.

**Figure 1:**
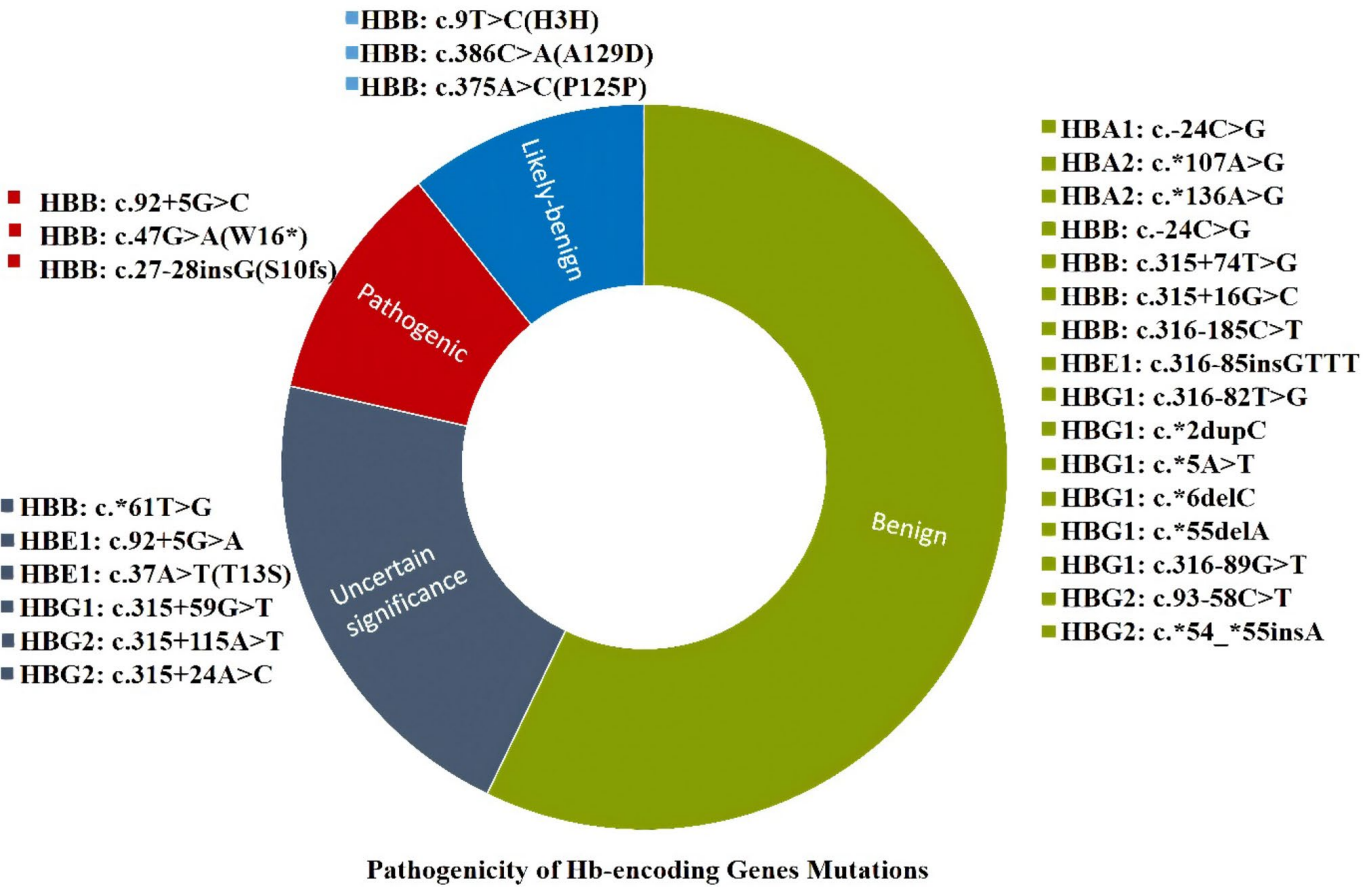
Pathogenicity prediction of detected Hb genes variants by Varsome: the green color label represents the benign variants, the red color shows the pathogenic variants, the blue color gives likely-benign and the gray color indicates variants of uncertain significance.

While many were anticipated as benign variants including HBA1:c.-24C>G, HBA2:c.*107A>G, HBA2: c.*136A>G, HBB: c.315+74T>G, HBB: c.315+16G>C, HBB: c.316-185C>T, HBE1: c.316-85insGTTT, HBG1: c.316-82T>G, HBG1: c.*2dupC, HBG1: c.*5A>T, HBG1: c.*6delC, HBG1: c.*55delA, HBG1: c.316-89G>T, HBG2: c.93-58C>T and HBG2: c.*54_*55insA. Three variants of HBB c.9T>C (p.H3H), c.386C>A(p.A129D) and c.375A>C(p.P125P) were classified as likely benign variants by Varsome. Moreover HBB: c.*61T>G, HBE1: c.92+5G>A, HBE1: c.37A>T (p.T13S), HBG1: c.315+59G>T, HBG2: c.315+115A>T and HBG2: c.315+24A>C were found to be variants of uncertain significance (Fig. 1).

### Molecular effect of pathogenic variants

Here we elucidated the molecular impact of pathogenic variants in the disease progression.

### HBB: c.47G>A (p.W16*) variant

The HBB: c.47G>A (p.W16*) is the coding sequence stop gain variant which was found to be associated with the β- thalassemia. This variant incorporates premature stop codon (amber) UAG at the 16th position resulting in the termination of translation and formation of truncated HBB protein. The mutant polypeptide consisting of 45 amino acid residue thus formed lacks the alpha helices (21–146 amino acid position), 2, 3- bisphosphoglycerate (2, 3-BPG) binding sites (at 83 and 144 amino acid position) and a metal binding site (at 64 and 93 position of amino acid). All these changes disrupt the binding of β-globin with the iron and thus reduce its oxygen binding property. This polypeptide also loses its ability to fold into globin protein and is unable to bind to the alpha chain resulting in the lack of Hb tetramer formation. Hence, this variant leads to the loss of β globin protein production and the Hb lacks the β globin subunit thus not able to function properly leading to β^0^ thalassemia.

### HBB: c.27-28insG (p.S10Vfs*14) variant

HBB pathogenic variant c.27-28insG (p.S10fs), also known as c.27dupG (S10Vfs*14), is a frameshift variant that involves the insertion/duplication of G nucleotide and leads to alter few essential amino acid (A11C, V12L, T13Y, A14C, L15P, W16V, G17G, K18Q, V19V, N20E, V21L, D22G) and formation of premature termination codon (opal) at 14th position in the downstream ORF. This variant leads to the formation of truncated protein due to the premature termination of translation. In accordance with the β globin domain, the protein encoded by this variant lacks important binding sites i-e two heme binding sites and two 2,3-bisphosphoglycerate binding sites, helix and non-glycated sites. Thus the encoded Hb protein lacks the β globin chain and is not able to transport oxygen. Moreover, this truncated mRNA may be subjected to nonsense-mediated mRNA decay, resulting in the loss of protein.

### HBB: c.92+5G>C variant

The HBB: c.92+5G>C is classified as an intronic pathogenic variant of HBB. Functional impact analysis showed that this variant losses the canonical splicing donor site which consequently leads to the loss of start codon (ATG) and excision of exon 1 from the open reading frame (ORF). Due to altered mRNA splicing pattern this variant encodes a shortened mRNA and may result in the loss of protein expression.

### Prediction of mutant proteins’ 3D structures

**Figure 2:**
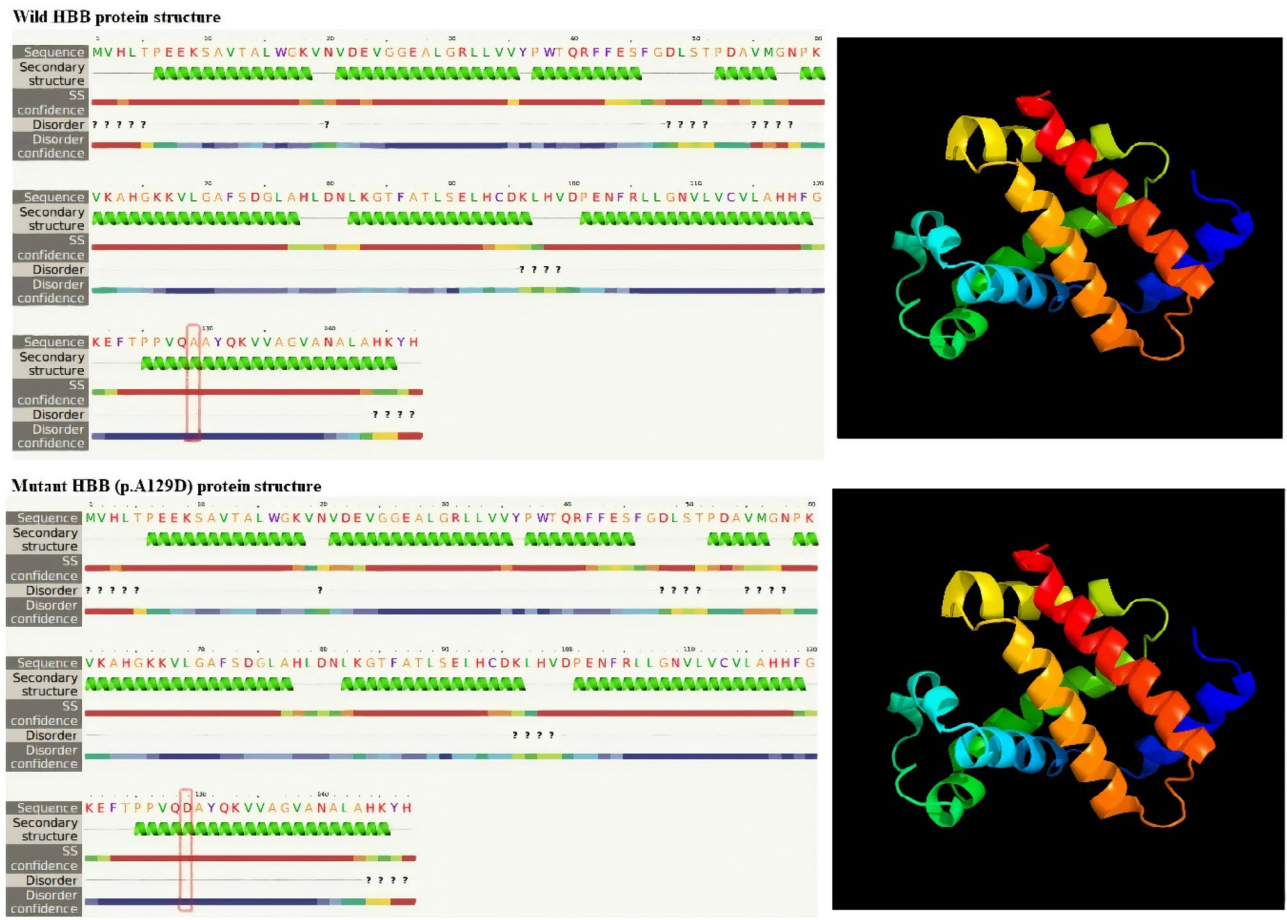
3D structure of β-globin protein: comparison of wild type β globin protein 3D and secondary structure with the mutant β globin (A129). The red-marked amino acid represents the position and the substituted amino acid. The Figure has been generated by Phyre2 tool version 2.0 (http://www.sbg.bio.ic.ac.uk/phyre2/html/page.cgi?id=index).

The secondary and 3D structure analysis of HBB: c.386C>A(p.A129D) variant protein showed that the variation lies in the alpha helix position at the tetramer interface region. But no major change in the 3D structure was observed (Fig. 2).

**Figure 3:**
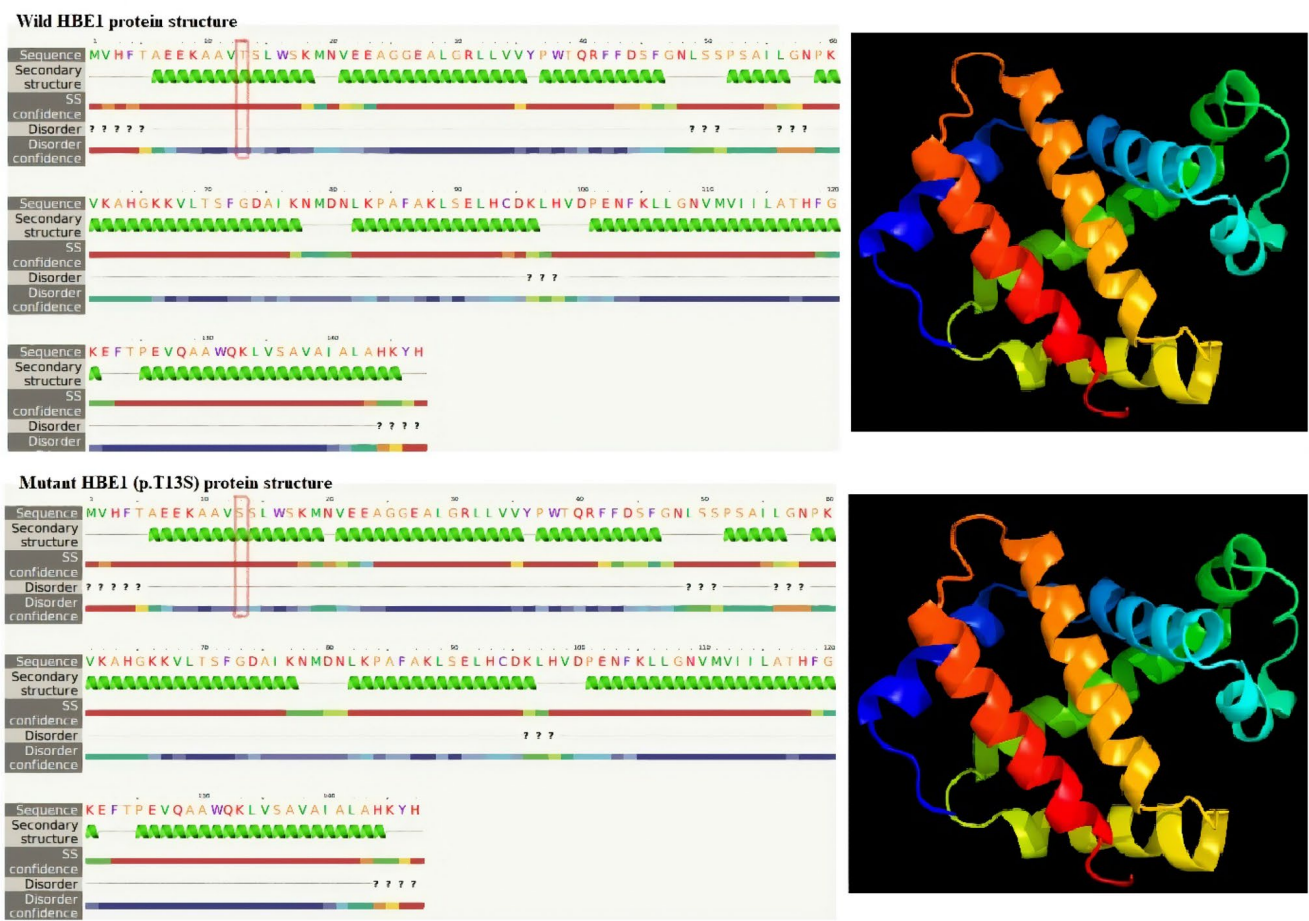
Structural effect of HBE1: c.37A>T(p.T13S) variant: Comparison of wild type protein 3D and secondary structure with the mutant protein. The red-marked amino acid represents the position and the substituted amino acid. The Figure has been generated by Phyre2 tool version 2.0 (http://www.sbg.bio.ic.ac.uk/phyre2/html/page.cgi?id=index).

While structural analysis of HBE1 variant c.37A>T(p.T13S) showed that the non-polar thymine present in the extracellular region is replaced by a non-polar serine residue in the alpha helix. This variation does not cause any possible effect on the secondary or 3D structure (Fig. 3).

## Discussion

Thalassemia is now considered a major health issue worldwide. In Pakistan, the inheritance rate of β-thalassemia has reached from 5.0 to 7.0%^6^. Defect in the Hb gene results in either the absence or inadequate synthesis of the alpha and β chain of Hb protein and the development of various types of thalassemia^17^. Therefore, there is a need to study the mutational spectrum of various Hb genes to explore the pattern and pathogenicity of Hb variants in the Pakistani population. In the current study, 49 families from Bahawalpur (South Punjab), Pakistan were screened for the detection of variants of different Hb genes including HBA1, HBA2, HBB, HBD, HBE1, HBG1 and HBG2.

Collectively via NGS analysis 20 variants were detected in patients and 20 variants were observed in carriers for all the Hb-encoding genes. Out of which 17 variants in patients and 17 variants found in carriers were observed to be the same. While 3 variants were observed specifically in thalassemia patients and 3 variants were present in only carriers. By Sanger sequencing only the 3 HBB variants (c.315+16G>C, c.315+74T>G and c.316-185C>T) were confirmed. Also, two novel variants of HBB (c.375A>C (p.P125P) and c.*61T>G) were discovered in the carriers. Moreover, in the HBE1 gene a novel variant HBE1: c.37A>T (p.T13S) and a unique variant (submitted in dbSNP but is reported for the first time in the Pakistani population) HBE1: c.92+5G>A were also detected by Sanger sequencing.

The β-globin gene variant HBB:c.92+5G>C observed in present study was known to be the most common (f = 45.0%) in a study of South Punjab thalassemia patients^18^. In another study, HBB:c.92+5G>C variant with an allelic frequency of 19% and the HBB: c.47G>A(p.W16*) with a 4.1% frequency were reported in the KPK region, Pakistan^6,19^. The HBB: c.47G>A(p.W16*) variant has also been observed in Saudi Arabians with 7.40% frequency^20^. The HBA2: c.*136A>G variant detected in our patients have not been reported earlier in Pakistani populations. But this variant was observed in Mediterranean alpha thalassemia patients^10^. The HBG1: c.316-89G>T variant (dbSNP ID: rs28379094) which we observed for the first time in Pakistanis is known as a frequently observed variant in all the other studied populations worldwide. The HBE1 missense variants c.37A>T resulting in the change in the amino acid threonine into serine (T13S) was neither detected in any population nor described earlier in any study globally thus is classified as a novel variant reported by our group. Moreover, a unique splice site variant HBE1: c.92+5(G>A) variant was noticed for the first time in the Pakistani population and was found not to be described earlier in any study worldwide but was noted in dbSNP database (rs1053824459). Above defined variants of HBA2, HBE1 and HBG1 might confer the thalassemia phenotype in the Pakistani population. Further study is required for conclusive evidence.

The variants detected in our carrier samples have been reported in the previous study in the fetus and the thalassemia patients in Pakistan and worldwide. However, these variants have also been examined in carriers worldwide but not reported in Pakistani carriers. The HBB: c.27_28insG (p. Ser10Valfs*14) variant detected in our carriers was observed in another study with (39.1%) frequency in the fetus during prenatal diagnosis in South Punjab and is considered as the second most observed variant (37.3%) in Pakistani thalassemia patients^18,21^. This variant is known to be among the most common variants detected in different provinces of Pakistan i.e., Punjab, Sindh and KPK with (31.70%) allele frequency^22^. It is reported as genetic indicator of thalassemia major fetal phenotype with frequency of 24.6%^23^ and used in the prenatal diagnosis of the Iranian population^24^. This variant was also reported in Saudi Arabians thalassemia patients with a 7.4% frequency^20^. The HBB: c.386C>A (p.A129D) variant uniquely reported in Pakistani carriers has also been observed earlier in a Japanese family^25^. HBB: c.375A>C (p.P125P) and HBB: c.*61T>G variants were discovered as novel variants. Two unique benign variants including HBA2: c.-24C>G and HBG2: c.*54_*55insA detected in carriers have not been noticed earlier in the Pakistani population. But, previously HBG2: c.*54_*55insA was identified internationally in the alpha-thalassemia carrier (f = 0.375), β-thalassemia carrier (f = 0.219) and β-thalassemia major patients with (f = 0.125) allelic frequency^26^.

The current study has also reported the presence of few HBB variants which were commonly present in both thalassemia patients and carriers. This study described 4 common variants of the HBB gene including c.9T>C (p.H3H), c.316-185C>T, c.315+74T>G and c.315+16G>C. In 2016 HBB: c.9T>C variant with 0.69 allele frequency was reported in Pakistani thalassemia patients^22^.While it was previously known as a rare β-thalassemia variant in the Saudi Arabia population^20^. The 3 HBB benign variants (c.316-185C>T, c.315+74T>G and c.315+16G>C) observed in our study were found in contradiction with the already reported variants in reference to the alteration in the nucleotide at these intronic positions. An earlier study^22^ has reported HBB: c.315+74G>T variant with 0.59 frequency and HBB:315+16C>G with 0.65 frequency. Another study^27^ has reported HBB:316-185T>C in Pakistani thalassemia patients. This nucleotide difference might be due to the framework polymorphism at these positions in normal sequence as described earlier^28^. These three HBB variants were also reported in thalassemia patients and carriers of Greek and other Mediterranean Populations^29^. In a previous study conducted by Moatter et al. on prenatal diagnosis of thalassemia, HBB: c.315+16C>G and HBB: c.315+74G>T were found as rare variants in fetal chorionic villus sample (CVS) and thus this approach cannot be applied for conventional CVS diagnosis in Pakistan^7^. In the Northern Iranian population HBB:c.315+74T>G (f = 54.71%) variant with the highest heterozygosity rate has been reported as a candidate for prenatal diagnosis of thalassemia^30^. The HBB: c.315+16G>C variant has been referred as SNP marker and used in non-invasive prenatal diagnosis (NIPD) in different populations. Moreover, it is associated with the detection of transfusion-dependent thalassemia (TDT) in the South Iranian population^31^. These findings suggest that the detected variants of the HBB gene can be further used as diagnostic marker in Pakistani patients.

In earlier studies, deletion of HBA genes including − α3.7, − α4.2, and ααα anti-3.7 alleles were identified in the Pakistani population^32^. Only one non-deletion variant HB Sallanches [a 104(G11) Cys+Tyr] of HBA was described in the Pakistani population^27^. But we have reported one non-deletional variant in the HBA1 gene common in carriers and patients. and three variants in the HBA2 gene. HBA1 benign variant c.-24C>G (rs374054030) was not noted earlier in the Pakistani population. This variant was observed to be frequent in South Asians. The sample having this c.-24C>G variant might be the sample with HBA12 conversion^33,34^. The HBA2: c.*107A>G variant is new to be reported in the Pakistani population but previously detected in Albanian patients^35^.

All the noted HBG1 variants were reported for the first time in Pakistani thalassemia patients and carriers. But these variants were previously reported globally in different thalassemia patients and carriers. As, HBG1: c.*55delA variant was reported internationally in alpha thalassemia carriers (f = 0.062), β-thalassemia carriers (f = 0.182) and delta-thalassemia (f = 0.116) patients^26^. The HBG1: c.316-82T>G was noted earlier in sickle-cell anemia patients^36^. Moreover, HBG1: c.*2dupC (rs371890964) HBG1: c.*6delC (rs3841756), HBG1: c.*5A>T (rs1065686) and HBG1: c.315+59G>T (rs33988501) variants were neither reported in any previous study nor in HbVar but found in dbSNP database. Another HBG2 unique variant c.315+115A>T was also reported in a previous study^37^. HBG2:c.315+24A>C and HBG2: c.93-58C>T were also reported internationally in an earlier study^26^. Further, the unique variant of HBE1: c.316-85insGTTT (rs3061750), has not been reported earlier in any study.

It is known that paternally inherited mutant alleles in the fetus will result in the development of a β-thalassemia carrier or a β-thalassemia major carrier fetus. The identification of common variants of hemoglobin variants in patients and carriers can define the inheritance pattern of these variants based on homozygosity or heterozygosity and thus can be used for the carrier screening and prenatal diagnosis of β-thalassemia purposes after the exploration of genetic lineage.

**Figure 4:**
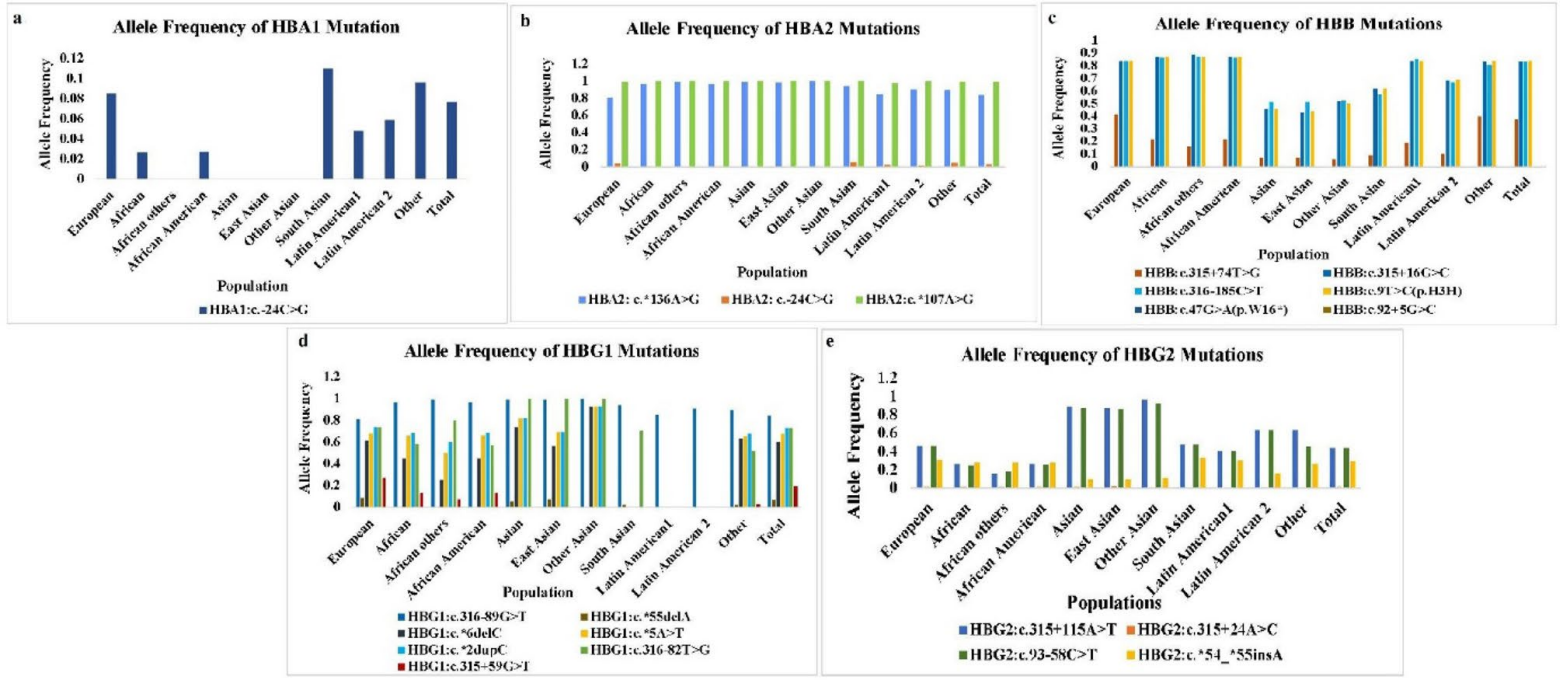
Allele frequency of hemoglobin encoding gene (HBA1, HBA2, HBB, HBG1, HBG2 and HBE1) in different populations all around the World. (**a**) Allele frequency of HBA1 variant (**b**) Allele frequency of HBA2 variants (**c**) Allele frequency of HBB variants (**d**) Allele frequency of HBG1 variants (**e**) Allele frequency of HBG2 variants. Colored bars represent the frequency of different Hb-genes variants**.** Variants with no allelic frequency in the graph are the rare variants observed in different populations. Allelic frequency of novel HBB: c.386C>A(p.A129D), HBB: c.375A>C(p.P125P), HBB: c.*61T>G and HBE1: c.37A>T(p.T13S) variants have not been provided by the dbSNP database.

Altered allele frequency (ALFA project) provided by dbSNP (https://www.ncbi.nlm.nih.gov/snp/) demonstrated the worldwide prevalence of all the detected variants (Fig. 4, Supplementary Tables S5–S9). The HBA1: c.-24C>G was found as a rare variant (f = 0.00) in Asian, East Asian, and African populations. The HBB variants (c.47G>A(p.W16*), c.92+5G>C), HBG2:c.315+24A>C, HBE1:(c.92+5(G>A), c.316-85insGTTT) variants were discovered to be rare (f = 0.00) around the globe. While HBG1 variants (c.*5A>T, c.*2dupC, c.315+59G>T and c.*6delC) were detected as rare variants in South Asians, Latin American (1, 2). Besides these, HBA2: (c.*136A>G, c.*107A>G), HBB (c.9T>C (p.H3H), c.316-185C>T, c.315+16G>C, c.315+74T>G), HBG1: c.316-89G>T, and HBG2 (c.315+115A>T, c.93-58C>T, c.*54_*55insT) variants were detected as worldwide common and frequent variants with highest frequency (f = 1, 1, 0.96 and 0.92) of HBA2: c.*136A>G, HBG1: c.316-89G>T, HBG2: c.315+115A>T and HBG2: c.93-58C>T respectively being noted in other Asians and f = 1 for HBA2: c.*107A>G in all the Asians populations. This spectrum of altered allele frequency describes the incidence of specific variants across different populations and helps to design the diagnostic strategy accordingly. The functional characterization of variants provided a great advantage in elucidating their part in disease progression and designing the suitable therapy. Previously, no study documented the functional impact of our most reported variants. Hence, in the current study, identified variants of Hb-encoding genes were further analyzed to evaluate their functional aspects and possible role in the progression of thalassemia. A total of 2 exonic HBB variants i.e. c.47G>A (p.W16*) and c.27-28insG (p.S10fs) were found to be pathogenic as predicted by the Varsome. Previously, Clinvar also classified these variants to be pathogenic for β^0^-thalassemia. The functional analysis of these pathogenic variants also revealed a deleterious impact on the protein function and structure and found to be disease-causing by MutationTaster. These pathogenic variants of HBB were examined to promote the premature termination of translation. Clinvar also reported the reduced or loss of protein expression due to these variations. The HBB (p.W16*) variant was found to alter the splicing of mRNA due to the alteration of the ESE/ESS motif ratio. The HBB: c.386 C>A (p.A129D) was classified as a likely-benign variant by Varsome. Previously, it was reported as a variant of unknown significance by Clinvar. Functional analysis of this variant showed it to be disease-causing and damaging by all the tools. The ithanet portal of hemoglobinopathies (https://www.ithanet.eu/) discloses the molecular effect of this variant which states that the substitution of Asp at 128 position in the 8^th^ helix of β globin chain leads to weakening the interaction between the alpha and β chain and inhibits the α1β1 dimer formation leading to the accumulation of free β globin protein. The effect of this variant on protein solubility also gives contrasting results. The HBB synonymous variant c.9T>C (p.H3H) classified as likely-benign by Varsome was indicated to be a neutral and benign variant having no potential effect on the protein function and structure by all the other functional analysis tools. An interesting situation was seen in the case of the HBB (p.P125P) variant. This variant was known to be neutral having no effect of amino acid substitution on the protein. But at the DNA level alteration of adenine nucleotide (A) into cytosine (C) at 375 position of coding sequence results in the activation of cryptic splice site affecting the splicing of mRNA and was predicted as a likely-benign variant. The non-synonymous variant of HBE1: c.37A>T (p.T13S) was predicted to be a variant of uncertain significance by Varsome. Functional analysis revealed this variant as neutral by all the tools discussed above except PANTHER which predicted a probably damaging effect of this variation on the protein.

Of all the detected intronic variants of HB encoding genes, only one HBB variant c.92+5G>C was identified to be pathogenic for the β^0^ thalassemia by Varsome. An investigation of the functional impact of this variant demonstrated that this variant broke the wild-type donor site resulting in the shortening of mRNA transcript thus affecting the protein formation. Clinvar also demonstrated that this variant may cause loss of protein expression. It was also known to be instigating for β+thalassemia in Pakistani populations in which a slight decrement in the synthesis of β-chains occurs resulting in mild to intense hepato-splenomegaly and patients show higher total HB alpha chain^38^. In East Asians and Asian Indians, this variant was found to be responsible for the β^0^ phenotype, in which no production of β-chains occurs^39^. The HBG2: c.315+24A>C variant resulted in the activation of a new acceptor splice site and thus predicted to alter the splicing pattern. All the other observed benign intronic variants (HBB: c.315+74T>G, HBB: c.315+16G>C, HBB: c.316-185C>T, HBE1: c.316-85insGTTT, HBG1: c.316-82T>G, HBG1: c.316-89G>T, and HBG2: c.93-58C>T) and variants of uncertain significance (HBE1:c.92+5G>A, HBG1: c.315+59G>T and HBG2: c.315+115A>T) showed no impact on the splicing mechanism.

All the observed UTR variants were benign variants except HBB: c.*61T>G novel variant which was predicted to be of uncertain significance. The analysis of the effect of 3′UTR variants on the miRNA binding sites revealed only HBG1: c.*55delA and HBG2: c.*54_*55insA variants are present in the miRNA target site but these variations do not alter binding affinities of corresponding miRNA. However, the functional study of these UTR variants by MutationTaster indicated that these variants altered the splicing pattern by the activation or loss of donor and acceptor splice sites and alteration of poly A signal. These findings suggest that functional characterization of variants is necessary for the identification of target variants to be used as a biomarker.

## Conclusion

To conclude all this, we have observed that necessarily not all the variants would be responsible for the disease progression. In this study, we have identified only three variants of β globin gene to be pathogenic and potentially damaging. These pathogenic variants can play a crucial role as biomarkers for designing better diagnostic and treatment strategies. The identified variants of uncertain significance can further be explored to elucidate their impact. Moreover, the variants observed to be common in patients and carriers can be used for diagnosis and carrier screening purposes but this will require further evaluation of genetic lineage.

### Supplementary Information

Supplementary Information.

## Data Availability

The datasets generated and/or analyzed during the current study are available in the European Variation Archive (EVA) repository, Project: PRJEB50808, Analyses: ERZ5128341, Web link: https://www.ebi.ac.uk/ena/browser/view/PRJEB50808..
