# Supplementary material for: Mutational analysis of hemoglobin genes and functional characterization of detected variants, through in-silico analysis, in Pakistani beta-thalassemia major patients
Source: Sci Rep. 2023 Aug 14;13:13236. doi: 10.1038/s41598-023-35481-1 (PMC10425424; doi:10.1038/s41598-023-35481-1)
Supplement: Supplementary file 1 — Supplementary Information. [file 41598_2023_35481_MOESM1_ESM.pdf]

Mutational Analysis of Hemoglobin Genes and Functional Characterization of Detected Variants, through *In-Silico* Analysis, in Pakistani Beta-Thalassemia Major Patients

Samina Ejaz[1†*], Iqra Abdullah[1†], Muhammad Usman[2†], Muhammad Arslan Iqbal[3], Sidra Munawar[1], Muhammad Irfan Khan[4], Nagina Imtiaz[2], Hanniah Tahir[1], Muhammad Ihsan Bari[1], Tayyaba Rasool[1], Aneeza Fatima[1], Ramsha Anwar[1], Ayman Durrani[2], Yasir Hameed[2]

[1]Department of Biochemistry, Institute of Biochemistry, Biotechnology and Bioinformatics (IBBB), The Islamia University of Bahawalpur, Bahawalpur (63100), Pakistan.

[2]Department of Biotechnology, Institute of Biochemistry, Biotechnology and Bioinformatics (IBBB), The Islamia University of Bahawalpur, Bahawalpur (63100), Pakistan.

[3]Punjab Institute of Neurosciences, Lahore, Pakistan.

[4]Department of Hematological Diseases, Thalassemia and Bone Marrow Transplantation, Bahawal Victoria Hospital, Bahawalpur (63100), Pakistan.

[*]Corresponding Author

Address correspondence to: Samina Ejaz, Department of Biochemistry, Institute of Biochemistry, Biotechnology and Bioinformatics (IBBB), The Islamia University of Bahawalpur, Bahawalpur (63100), Pakistan. Phone: 092 301 6812346, E-mail: samina.ejazsyed@iub.edu.pk, saminaejazsyed@yahoo.com

[†]These authors have contributed equally to this work and share first authorship

Supplementary Data Tables:

Supplementary Tables 1: Demographic and clinical detailed of enrolled thalassemia patients

Sr.no.	Demographic information		Patients number
1	Gender	Male	28
		Female	21
2	Age	6-9 months	3
		1 year	2
		1.5 year	5
		2.5 year	2
		3 year	4
		3.5 year	1
		4 year	2
		5 year	7
		6 year	5
		6.5 year	1
		7 year	4
		8 year	2
		8.5 year	2
		9 year	2
		10 year	3
		13 year	2
		14 year	1
		15 year	1
**Clinical information**			
1	Hemoglobin Measurements	**Measurements in Patients (Mean±SD)**	**Normal reference value**
	Hb (g/dL)	5.6±1.8	>7
	HbA (%)	83.1±24.8	96.8-97.8
	HbA2 (%)	2.6±1.1	2.2-3.2
	HbF (%)	40.7±40.60	<0.5
2	Hematology measurements		
	MCV (fL)	70.01±7.0	76-96
	MCH (pg)	23.5±2.9	27-32

3	Blood group	A+	7
		B+	10
		B-	2
		AB+	1
		O+	11
		O-	1
		Undefined	17
4	Transfusion frequency (days)	20	1
		15	2
		30	46
5	Age of thalassemia diagnosis	At birth age	15
		2-8 months age	18
		1-2.5 years age	6
		At 4 year age	1
		At 5 year age	1
6	Spleen surgery	None	
7	Iron chelation therapy (%)	20(Oral)	

Supplementary Tables 2: Demographic details of carriers included in the present study.

Sr.No.	Demographic information		Number of carriers
1	Gender	Male	31
		Female	18
2	Age (year)	30-35	28
		36-40	16
		41-45	5
		Bahawalpur	14
		Lodhran	5
		Ahmadpur	10
		Malsi	2
		Yazman	2
		Liaqatpur	2
		Uch Shareef	2

3	Residence	Kahrorpakka	4
		Dunyapur	1
		Haroonabad	1
		Dunyapur	1
		Multan	1
		Hasilpur	1
		Shahadra	1
		Khairpur	1
		Bahawalnagar	1
4	Merital Status	Cousin Marriages	38
		Others	11

Clinical information		
1	**Hemoglobin Measurements**	**Measurements in Carriers**
	HbA2 (%)	4
	HbF (%)	4.5
	**Hematology measurements**	
	MCV (fL)	<80
2	MCH (pg)	<27

Supplementary Table 3: Detailed profile of thalassemia major patients included in the present study

Patients ID	Gender	Age	Transfusion frequency	Blood group	Age of diagnosis	Residence	Parents merital status
P1	Male	6 months	30 days	B+	At birth	Bahawalpur	Cousin marriage
P2	Female	7 months	30 days	B+	At 4 month	Yazman	Cousin marriage
P3	Male	9 months	30 days	B+	At 8 months	Liaqat pur	Cousin marriage
P4	Male	1 year	30 days	O+	At 4 month	Uch Shareef	Cousin marriage
P5	Female	1 year	30 days	O+	At 4 months	Bahawalpur	NCM
P6	Male	1.5 year	30 days	B+	At 7 month	Uch Shareef	Cousin marriage
P7	Female	1.5 year	30 days	B+	At 4 months	Bahawalpur	NCM
P8	Male	1.5 year	30 days	O+	At 5 months	Malsi	Cousin marriage
P9	Female	1.5 year	30 days	O+	At 5 months	Malsi	NCM
P10	Male	1.5 year	30 days	A+	At 5 months	Ahmadpur	Cousin marriage
P11	Male	2.5 year	30 days	-	-	Ahmadpur	Cousin marriage
P12	Male	2.5 year	30 days	O+	At birth	Lodhran	Cousin marriage
P13	Male	3 year	30 days	O+	At birth	Ahmadpur	NCM
P14	Male	3 year	30 days	-	-	Ahmadpur	NCM
P15	Male	3 year	30 days	O+	At birth	Bahawalpur	NCM
P16	Male	3 year	30 days	A+	-	Bahawalpur	Cousin marriage

P17	Male	3.5 year	30 days	A+	-	Ahmadpur	Cousin marriage
P18	Male	4 year	30 days	B-	At birth	Bahawalpur	Cousin marriage
P19	Male	4 year	20 days	-	3 months	Kahrorpakka	Cousin marriage
P20	Male	5 year	30 days	-	5 months	Kahrorpakka	Cousin marriage
P21	Female	5 year	30 days	-	At birth	Ahmadpur	Cousin marriage
P22	Female	5 year	30 days	-	At birth	Lodhran	Cousin marriage
P23	Male	5 year	30 days	B+	-	Haroonabad	Cousin marriage
P24	Female	5 year	30 days	B-	4 year	Bahawalpur	Cousin marriage
P25	Male	5 year	30 days	O+	6 months	Lodhran	Cousin marriage
P26	Male	5 year	30 days	-	1 year age	Kahrorpakka	Cousin marriage
P27	Female	6 year	30 days	-	-	Bahawalpur	NCM
P28	Female	6 year	30 days	O+	3 months	Dunyapure	Cousin marriage
P29	Female	6 year	15 days	B+	at birth	Multan	Cousin marriage
P30	Male	6 year	30 days	B+	At 1.5 year	Bahawalpur	Cousin marriage
			30 days	-	-	-	Cousin marriage
P31	Female	6 year	30 days	B+	At birth	Kahrorpakka	Cousin marriage
P32	Female	6.5 year	15 days	O+	At 1.5 year	Bahawalpur	Cousin marriage
P33	Female	7 year	30 days	B+	At 2 year	Yazman	Cousin marriage
P34	Male	7 year	30 days	A+	At 6 month	Ahmadpur	Cousin marriage
P35	Male	7 year	30 days	-	At birth	Hasilpur	Cousin marriage
P36	Female	7 year	30 days	-	2.5 months	Ahmadpur	Cousin marriage

P37	Male	8 year	30 days	O+	At birth	Lodhran	Cousin marriage
P38	Female	8 year	30 days	A+	At birth	Liaqatpur	Cousin marriage
P39	Male	8.5 year	30 days	AB+	At birth	Bahawalpur	NCM
P40	Male	8.5 year	30 days	-	At birth age	Ahmadpur	NCM
P41	Female	9 year	30 days	-	At birth age	Khairpur	NCM
P42	Male	9 year	30 days	-	At 1.5 year	Lodhran	NCM
P43	Male	10 year	30 days	-	At 1 year	Bahawalpur	Cousin marriage
P44	Female	10 year	30 days	-	At 6 month	Bahawalnagar	Cousin marriage
P45	Male	10 year	30 days	O-		Ahmadpur	Cousin marriage
P46	Female	13 year	30 days	A+	At birth	Bahawalpur	Cousin marriage
P47	Female	13 year	30 days	-	At 3 month	Shahadra	Cousin marriage
P48	Female	14 year	30 days	-	At 5 month	-	Cousin marriage
P49	Female	15 year	30 days	A+	At 5 year age	Bahawalpur	NCM

NCM= Not cousin marriage
"-" Donates not identified

Supplementary Table 4: List of HB encoding genes primers used in the present study

Sr. No.	Genes	Primer ID	Primer sequence	Product size (bp)	Annealing temperature (℃)
1	HBA1	F1	AGGGTGGAGACGTCCTGG	759	60
		R1	AGAAGAGGGTCAGTGGGGCCGAG		
		F2	GCCCCAAGCATAAACCCTGG	640	
		R2	AGAAGAGGGTCAGTGGGGCCGAG		
		F3	ACAGGCCACCCTCAACCGTCC	670	
		R3	ATGCCTGGCACGTTTGCTGAGG		
		F4	CACCACCAAGACCTACTTCC	588	
		R4	ATGCCTGGCACGTTTGCTG		
2	HBA2	F1	AGGGTGGAGACGTCCTGG	788	60-62
		R1	AGAGAAGAGGGTCAGTGC		
		F2	CCCAAGCATAAACCCTGG	624	
		R2	AGAGAAGAGGGTCAGTGC		
		F3	ACAGGCCACCCTCAACCGTCC	701	
		R3	CCATTGTTGGCACATTCC		
		F4	CACCACCAAGACCTACTTCC	648	
		R4	AGAGGTCCTTGGTCTGAGACAGG		
3	HBB	F1	CCAATCTACTCCCAGGAGCA	323	55
		R1	GGCAGAGAGAGTCAGTGCCTA		
		F2	GAAGACTCTTGGGTTTCTGA	404	
		R2	TCATTCGTCTGTTTCCCATTCTAAAC		
		F3	GTGTACACATATTGACCAAATCAGGGTA	293	
		R3	GGTAGCTGGATTGTAGCTGC		
		F4	CTGGATTATTCTGAGTCCAAGC	309	
		R4	ATTAGGCAGAATCCAGATGCTC		
4	HBD	F1	ACTAATGAAACCCTGCTTATC	298	55

		R1	TGTCTACACATGCCCAGTTT		
		F2	GAAAAGTGAAGCATCTCCTGG	275	
		R2	AATATCCTGTCTTTCTCTCCCAAC		
		F3	AATATCCTGTCTTTCTCTCCCAAC	133	
		R3	TAATTTCTGCTCTTTGGAGGTAG		
		F4	GGGCAAGTTAAGGGAATA	718	
		R4	GGAGAAGAGCAGGTAGGT		
5	HBE1	F1	GGTGCTGACTTCCTTTGGAG	599	60
		R1	TGTGACCAAGTGAAGGCTTG		
		F2	TGTGACCAAGTGAAGGCTTG	600	
		R2	TCAGGTGCTGGTGATGTGAT		
		F3	GGCACAAACCTTGGAACAGT	169	
		R3	CTCCTGGGTAACGTGATGGT		
		F4	ACTGGTAAAATGGGGAAGGG	160	
		R4	TGTGACCAAGTGAAGGCTTG		
6	HBG1	F1	AAACGGTCCCTGGCTAAACT	533	53
		R1	GGAAGTCAGCACCTTCTTGC		
		F2	CCCTTCCCCACACTATCTCA	590	
		R2	GGAAGTCAGCACCTTCTTGC		
		F3	GAAGCACCCTTCAGCAGTTC	551	
		R3	ACTAAAGGCAACAGGGCTGA		
		F4	GCAAGAAGGTGCTGACTTCC	529	
		R4	ATGTTTGGCCACCAAAGTTC		
7	HBG2	F1	TGAATGTGGAAGATGCTGGA	547	56
		R1	AAACATTGCCACTGGGTCTC		
		F2	CTTCCTTGGGAGATGCCATA	518	
		R2	ATGTTTGGCCACCAAAGTTC		
		F3	CTGCACTGTGACAAGCTGCAT	971	
		R3	CGAAATGGATTGCCAAAACG		

Supplementary Table 5: Worldwide allele frequency of HBA1 variants

**Populations**	**Mutation**	
	**HBA1:c.-24C>G**	
	**Sample Size**	**Frequency**
European	18142	0.08516
African	3410	0.0264
African others	116	0
African American	3294	0.0273
Asian	164	0
East Asian	110	0
Other Asian	54	0
South Asian	94	0.11
Latin American1	146	0.048
Latin American 2	610	0.059
Other	2754	0.0966
Total	25320	0.07717

Supplementary Table 6: Worldwide allele frequency of HBA2 variants

**Populations**	**Variants**					
	**HBA2: c.*136A>G**		**HBA2: c.-24C>G**		**HBA2:c.*107A>G**	
	**Sample Size**	**Frequency**	**Sample Size**	**Frequency**	**Sample Size**	**Frequency**
European	14152	0.80921	8682	0.0438	14318	0.99378
African	2898	0.9689	2882	0.0087	2978	0.9976
African others	114	0.991	110	0	114	1
African American	2784	0.968	2772	0.009	2864	0.9976
Asian	112	0.991	132	0	116	1
East Asian	86	0.99	96	0	88	1
Other Asian	26	1	36	0	28	1
South Asian	98	0.94	94	0.06	98	1
Latin American1	146	0.849	146	0.027	154	0.974
Latin American 2	610	0.905	610	0.018	616	0.997
Other	504	0.897	870	0.052	954	0.992
Total	18520	0.84185	13416	0.03511	19234	0.99428

Supplementary Table 7: Worldwide allele frequency of HBB variants

Populations	Variants											
	HBB:c.315+74T>G		HBB:c.315+16G>C		HBB:c.316-185C>T		HBB:c.9T>C(p.H3H)		HBB:c.47G>A(p.W16*)		HBB:c.92+5G>C	
	Sample Size	Frequency	Sample Size	Frequency	Sample Size	Frequency	Sample Size	Frequency	Sample Size	Frequency	Sample Size	Frequency
European	12532	0.41031	19756	0.8361	125952	0.8392	43324	0.84087	13094	0.00015	90194	0.00002
African	2312	0.2137	3050	0.8738	9734	0.8626	3574	0.8721	2720	0	5402	0
African others	94	0.16	98	0.89	324	0.877	122	0.869	84	0	180	0
African American	2218	0.216	2952	0.8733	9410	0.8622	3452	0.8722	2636	0	5222	0
Asian	84	0.07	168	0.458	666	0.518	168	0.458	108	0	642	0
East Asian	68	0.07	112	0.429	534	0.517	112	0.438	84	0	488	0
Other Asian	16	0.06	56	0.52	132	0.523	56	0.5	24	0	154	0
South Asian	46	0.09	98	0.62	192	0.573	98	0.62	94	0	150	0
Latin American1	58	0.19	146	0.836	752	0.855	498	0.839	146	0	486	0
Latin American 2	282	0.099	610	0.685	6314	0.6691	628	0.688	610	0	2138	0
Other	722	0.398	2974	0.8315	6322	0.8086	7318	0.8416	768	0	5214	0.00002
Total	16036	0.37241	28802	0.8333	149932	0.8306	55608	0.8397	17540	0.00017	104226	0.000029

Supplementary Table 8: Worldwide allele frequency of HBG1 variants

Population	Variants													
	HBG1:c.316-89G>T		HBG1:c.*55delA		HBG1:c.*6delC		HBG1:c.*5A>T		HBG1:c.*2dupC		HBG1:c.316-82T>G		HBG1:c.315+59G>T	
	Sample Size	Frequency	Sample Size	Frequency	Sample Size	Frequency	Sample Size	Frequency	Sample Size	Frequency	Sample Size	Frequency	Sampl e Size	Frequency
European	6660	0.8092	12176	0.0834	12114	0.6121	10886	0.68133	16656	0.73733	6846	0.7371	1860	0.271
African	626	0.9689	2565	0.0008	932	0.445	632	0.657	608	0.686	118	0.585	1612	0.1272
African others	36	0.991	108	0	20	0.25	10	0.5	8	0.6	8	0.8	82	0.07
African American	590	0.968	2548	0.0008	912	0.45	622	0.659	600	0.687	110	0.573	1530	0.1301
Asian	4	0.991	40	0.05	62	0.74	56	0.82	56	0.82	4	1	2	0
East Asian	4	0.99	30	0.07	32	0.56	26	0.69	26	0.69	2	1	2	0
Other Asian	0	1	10	0	30	0.93	30	0.93	30	0.93	2	1	0	0
South Asian	24	0.94	60	0.02	20	0	2	0	0	0	6	0.7	16	0
Latin American1	42	0.849	112	0	27	0	4	0	0	0	4	0	42	0
Latin American 2	78	0.905	402	0	111	0	22	0	0	0	24	0	66	0
Other	146	0.897	404	0.022	2365	0.6381	2310	0.6537	2542	0.6735	52	0.52	118	0.025
Total	7580	0.8419	15850	0.0649	1561	0.60041	13912	0.67481	19862	0.72782	7054	0.7301	3716	0.1916

Supplementary Table 9: Worldwide allele frequency of HBG2 gene variants:

Populations	Variants							
	HBG2:c.315+115A>T		HBG2:c.315+24A>C		HBG2:c.93-58C>T		HBG2:c.*54_*55insA	
	Sample Size	Frequency	Sample Size	Frequency	Sample Size	Frequency	Sample Size	Frequency
European	12080	0.46084	18142	0.01262	14152	0.46064	4846	0.30305
African	2816	0.2617	3410	0.0114	2898	0.2457	38	0.2784
African others	108	0.157	116	0.009	114	0.175	2	0.279
African American	2708	0.2659	3294	0.0115	2784	0.2486	36	0.2784
Asian	108	0.889	164	0.012	112	0.875	0	0.095
East Asian	84	0.87	110	0.018	86	0.86	0	0.089
Other Asian	24	0.96	54	0	26	0.92	0	0.11
South Asian	94	0.47	94	0	98	0.47	2	0.33
Latin American1	146	0.404	146	0	146	0.404	4	0.301
Latin American 2	610	0.636	610	0.007	610	0.636	24	0.156
Other	478	0.636	2754	0.0054	504	0.45	32	0.2658
Total	478	0.43534	25320	0.01141	18520	0.43461	4946	0.2917
